# Sheehan syndrome with reversible dilated cardiomyopathy

**DOI:** 10.4103/0256-4947.65269

**Published:** 2010

**Authors:** Bashir A. Laway, Mohammad S. Alai, Tariq Gojwari, Mohd A. Ganie, Abdul H Zargar

**Affiliations:** a Department of Endocrinology, Sher-I-Kashmir Institute of Medical Sciences, Srinagar, Jammu and Kashmir, India; b Department of Cardiology, Sher-I-Kashmir Institute of Medical Sciences, Srinagar, Jammu and Kashmir, India; c Department of Radio Diagnosis, Sher-I-Kashmir Institute of Medical Sciences, Srinagar, Jammu and Kashmir, India

## Abstract

Cardiac abnormalities in patients with Sheehan syndrome are uncommon. A case of Sheehan syndrome with dilated cardiomyopathy is presented in whom hormone replacement with levothyroxine and prednisolone resulted in complete recovery of cardiomyopathy. A 25-year-old woman presented with lactation failure, secondary amenorrhea, features of hypothyroidism and a hypocortisol state following severe postpartum hemorrhage after her last child birth. She also had smear positive pulmonary tuberculosis. After starting antitubercular treatment, she developed shock, suggestive of hypocortisol crisis. Hormonal investigations revealed evidence of panhypopitutarism and magnetic resonance imaging revealed partial empty sella. Meanwhile echocardiography revealed evidence of dilated cardiomyopathy (DCM). The patient was given replacement therapy in the form of glucocorticoids and levothyroxine in addition to antitubercular treatment. She improved and on follow-up over a period of 7 months, the DCM completely reversed. To our knowledge this is the first report of reversible DCM in a patient with Sheehan syndrome.

Sheehan syndrome is the occurrence of panhypopitutarism following postpartum hemorrhage (PPH).^1^ It manifests with lactation failure, amenorrhea, involution of breasts, loss of axillary and public hair and features of other pituitary hormone deficiencies.^2^,^3^ The cause of panhypopitutarism is thought to be ischemic necrosis of the anterior pituitary secondary to postpartum hemorrhage.^4^ Because the syndrome may manifest long after the delivery, autoimmunity may play a role.^5^ Cardiac abnormalities have been reported in patients with hypopituitarism, most of these being linked to growth hormone deficiency.^6^–^8^ Detailed studies on cardiac function in patients with Sheehan syndrome are not available due to the rarity of the syndrome in developed nations. We report a case of Sheehan syndrome with concomitant pulmonary tuberculosis and dilated cardiomyopathy (DCM) that completely reversed with treatment. We believe this is the first such case reported in the literature.

## CASE

**Figure 1:**
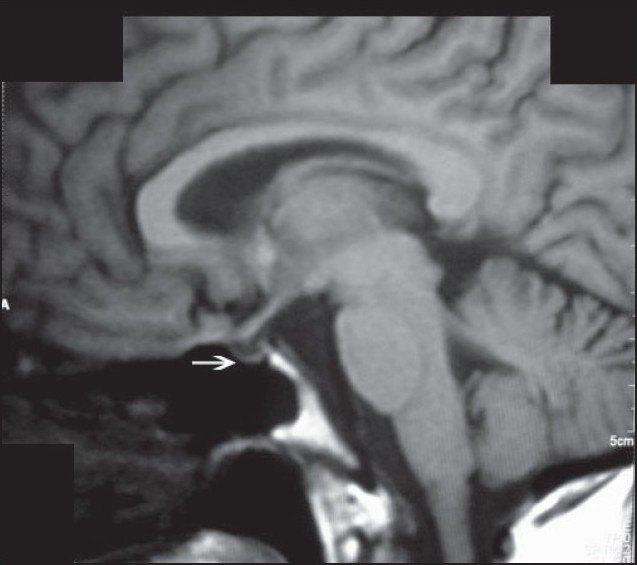
T1 weighted MRI pituitary, sagital view showing pituitary fossa filled with cerebrospinal fluid with stalk touching the base against a thin rim of compressed gland suggestive of an empty sella (arrow).

**Figure 2:**
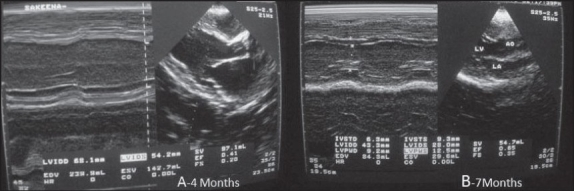
Echocardiographic left parasternal long axis view (2D and M mode) showing left ventricular dimensions and ejection fraction at four (A) and seven months (B) of treatment.

A 22-year-old woman underwent a lower segment cesarean delivery for her fourth pregnancy two years before; she was transfused two units of blood for continuous vaginal bleeding. After delivery she failed to lactate, did not resume cycles and complained of fatigue. Three months before admission she complained of cough and intermittent fever with night sweats. She was seen at a primary health center where sputum was found positive for acid fast bacillus on multiple occasions, with no findings suggestive of tuberculosis on chest radiograph. The patient was started on antiitubercular treatment (ATT) in the form of isoniazid, rifampicin, ethambutol and pyrazinamide in appropriate doses. She felt more fatigue and weakness in the first week after starting ATT. On the tenth day she was found to be unconscious in the morning and was brought to the hospital. Examination in emergency revealed a sick, pale looking lady with a feeble pulse rate of 100 beats per minute and unrecordible blood pressure with a temperature of 99°F. She had breast atrophy and sparse axillary and pubic hair. She also had a systolic murmur at the mitral area suggestive of mitral regurgitation. Her deep tendon jerks revealed a marked delay in relaxation suggestive of hypothyroidism. In view of her postpartum hemorrhage (and need for blood transfusions), lactation failure, secondary amenorrhea, hypotension and features of hypothyroidism, a clinical diagnosis of Sheehan syndrome with adrenal crisis was made. The patient was given emergency treatment in the form of oxygen inhalation, intravenous fluids and a nasogastric tube was inserted. After taking a blood sample she was started on hydrocortisone 100 mg every 6 hours, levothyroxine 100 μg through the NG tube and ATT was continued. She regained consciousness after an hour and her blood pressure rose to 80/60 mm Hg. Investigations revealed a hemoglobin of 9.2 g/dL, total leukocyte count of 3.2×10^3^/mL with polymorphonuclear leucocytosis, a platelet count of 64×10^3^/mL and a peripheral film suggestive of normocytic normochromic anemia. ECG revealed a heart rate of 90/min with low voltage complexes; chest X-ray revealed a cardiothoracic ratio of 0.5; the rest of the lung parenchyma was apparently normal. She had plasma glucose of 73 mg/dL. Her serum urea, creatinine, calcium, phosphorus, alkaline phosphatase, creatine kinase and lactate dehydrogenase were within normal limits. She had a mild increase in liver enzymes about twice the upper limit of normal secondary to ATT that remained stable throughout the entire hospital stay. Hormonal investigations revealed evidence of central hypothyroidism, hypogonadism, low prolactin, cortisol and growth hormone. All the hormonal estimations were performed with specific radioimmunoassay (Table 1). Plain T1 weighted magnetic resonance imaging (MRI) of the pituitary revealed evidence of a partial empty sella (Figure 1). Echocardiography revealed moderate-to-severe mitral regurgitation, a dilated left ventricle with severe diastolic dysfunction and global hypokinesia (initial ejection fraction of 23%), suggestive of DCM with severe left ventricular dysfunction. The patient improved and was discharged on levothyroxine 100 mg/day and prednisolone 7.5 mg/day, in addition to ATT. She was seen on follow-up after 4 months when she was clinically euthyroid and eucortisolemic. Echocardiography revealed marked improvement at 4 and 7 months (Figure 2) and at 7 months of follow up there was complete resolution of cardiac abnormalities (Table 2). The patient was again admitted 16 months after for review because of development of weight gain. Thyroxine was stopped for 6 weeks and prednisolone for 48 hours. Hormone investigations revealed serum total T4 of 1.60 μg/dL (normal value of 5.5-13.5), TSH of 1.26 μIU/mL (normal value of 0.2-5) and morning (8AM) cortisol of less than 0.1mg/dL (normal value of 12-25).

**Table 1:** Baseline plasma hormone analysis at admission.

Basal hormone	Value	Normal value
T3 (ng/mL)	<0.5	0.7-2.5
T4 (μg/dL)	1.8	5.5-13.5
TSH (mIU/mL)	0.39	0.2-5
LH (mIU/mL)	0.74	3-12
FSH (mIU/mL)	2.99	5-20
Prolactin (ng/mL)	<2.8	3-24
Cortisol (μg/dL)	5.8	12-25
GH (ng/mL)	<0.25	0-5

T3: tri iodothyronine; T4: thyroxine; TSH: thyroid stimulating hormone; LH: luteinizing hormone; FSH: follicle stimulating hormone; GH: growth hormone.

**Table 2:** Serial echocardiographic findings at admission, and 4 and 7 months after treatment.

Parameters	At admission	At 4 months	At 7 months
LVIDD (cm)	5.9	6.8	4.3
LVISD (cm)	4.8	5.4	2.8
EDV (mL)	201	238	84
ESV (mL)	119	142	29.6
EPSS (cm)	1.4	1.6	1
EF (%)	23	41	65

LVIDD: left ventricular internal diastolic diameter; LVISD: left ventricular internal systolic diameter; EDV: end diastolic volume; ESV: end systolic volume; EPSS: E point septal separation; EF: ejection fraction.

## DISCUSSION

Our patient had clinical and hormonal evidence of hypopituitarism following PPH in a clinical setting of postpartum pituitary necrosis. She was admitted in adrenal crisis after receiving ATT for a week. In addition, she had radiological evidence of an empty sella. During the evaluation for adrenal crisis she was found to have mitral regurgitation and echocardiographic evidence of DCM with a markedly decreased ejection fraction. She gradually improved with correction of hypocortisol and the hypothyroid state and cardiac abnormalities completely reversed after achieving euthyroid and eucortisolemic state. Cardiac abnormalities have been studied extensively in patients with primary hypothyroidism, almost one-third of patients have pericardial effusion, which disappears with correction of the hypothyroid state. Various types of cardiomyopathies, dilated or hypertrophic have been reported that reverse with replacement of levothyroxine.^9^,^10^ Cardiac abnormalities have also been reported in patients with hypopituitarism. Some of these abnormalities have been attributed to growth hormone deficiency, and in some reports, reversibility of cardiac failure has been reported with growth hormone replacement.^6^–^8^ Oki et al reported a case of ampulla cardiomyopathy in a patient with adrenal insufficiency and hypothyroidism secondary to pituitary adenoma. This patient also had severe hyponatremia secondary to hypopituitarism. Administration of hydrocortisone, thyroxine and hypertonic saline normalized the left ventricular wall motion abnormalities in 2 weeks. The possibility of catecholamine excess was presumed to be the cause of cardiomyopathy although the plasma levels of epinephrine, norepinephrine and dopamine were normal.^11^ Shah et al reported a middle aged woman with type 1 diabetes and hypertension with acute heart failure and neurological signs, investigations revealed central hypothyroidism. Echocardiography revealed a thickened myocardium suggestive of cardiomyopathy and subsequent coronary angiography was normal. There was complete resolution of cardiomyopathy following thyroxine treatment. Cardiomyopathy was presumed to be due to hypopituitarism although details of other pituitary hormones were not available and pituitary MRI was reported as normal.^12^ Parikh et al reported on a 37-year-old woman who concomitantly presented with lymphocytic hypophysitis, panhypopitutarism, postpartum cardiomyopathy and pneumonitis. Patient was initially put on high doses of prednisolone, thyroxine as well as angiotensin converting enzyme inhibitors and beta blockers. Over a period of 2 years the patient responded clinically and was maintained on replacement doses of prednisolone in addition to other medications. The woman was also found to have growth hormone deficiency, which was not replaced.^13^

There has been evidence that Sheehan syndrome has an autoimmune basis and an autoimmune etiology has also been suggested for cardiomyopathy.^5^,^14^ Whether the components of the syndrome also have an autoimmune basis is a possibility that needs to be studied in future.

Although cases of cardiomyopathy associated with hypopituitarism are known, no previous concomitant presence of Sheehan syndrome and cardiomyopathy has been reported in the literature. Our case has many atypical features that are relevant in this part of the world. This patient had cardiomyopathy at presentation, and recovered with levothyroxine and glucocorticoids without receiving any growth hormone. We believe the cause of DCM in this patient was either a hypothyroid and/or a hypocortisol state because it completely reversed with replacement of these two hormones. We do not think growth hormone deficiency was the cause as it was not replaced in this patient. In countries such as India, where Sheehan syndrome is quite common, it is worth getting an echocardiography at the time of diagnosis.
